# Association of differentially expressed *R*-gene candidates with leaf spot resistance in peanut (*Arachis hypogaea* L.)

**DOI:** 10.1007/s11033-020-06049-3

**Published:** 2021-01-05

**Authors:** Phat M. Dang, Marshall C. Lamb, Charles Y. Chen

**Affiliations:** 1 National Peanut Research Laboratory, United States Department of Agriculture-Agricultural Research Service, Dawson, GA USA; 2 Department of Crop, Soil and Environmental Sciences, Auburn University, Auburn, AL USA

**Keywords:** Resistance genes, *R*-genes, Cultivated peanut, Disease resistance, Leaf spot, Breeding lines, Gene-expression

## Abstract

Early leaf spot (ELS) and late leaf spot (LLS) are major fungal diseases of peanut that can severely reduce yield and quality. Development of acceptable genetic resistance has been difficult due to a strong environmental component and many major and minor QTLs. Resistance genes (*R*-genes) are an important component of plant immune system and have been identified in peanut. Association of specific *R*-genes to leaf spot resistance will provide molecular targets for marker-assisted breeding strategies. In this study, advanced breeding lines from different pedigrees were evaluated for leaf spot resistance and 76 candidate *R*-genes expression study was applied to susceptible and resistant lines. Thirty-six *R*-genes were differentially expressed and significantly correlated with resistant lines, of which a majority are receptor like kinases (RLKs) and receptor like proteins (RLPs) that sense the presence of pathogen at the cell surface and initiate protection response. The largest group was receptor-like cytoplasmic kinases (RLCKs) VII that are involved in pattern-triggered kinase signaling resulting in the production reactive oxygen species (ROS). Four *R*-genes were homologous to TMV resistant protein N which has shown to confer resistance against tobacco mosaic virus (TMV). When mapped to peanut genomes, 36 *R*-genes were represented in most chromosomes except for A09 and B09. Low levels of gene-expression in resistant lines suggest expression is tightly controlled to balance the cost of *R*-gene expression to plant productively. Identification and association of *R*-genes involved in leaf spot resistance will facilitate genetic selection of leaf spot resistant lines with good agronomic traits.

**Supplementary Information:**

The online version contains supplementary material available at 10.1007/s11033-020-06049-3.

## Introduction

Peanut (*Arachis hypogaea* L.) is an important source of nutritious food and oil [1], grown around the world in warm climates. United States (US) is the 4th largest peanut producers in the world, following China, India, and Nigeria, producing around 6% (5.5 million metric tons) of world’s peanut production [2]. The Southeast region (Georgia, Florida, Alabama, Mississippi) produces the highest percentage of all US grown peanuts at 68%, followed by the Southwest region (Texas, Oklahoma, New Mexico) at 18%, and the Virginia-Carolina region at 13% (American Peanut Council). Among the four market types in the US, runner-type contributes 85% of the total peanut production and is grown mostly in Georgia, Alabama, and Florida, followed by Virginia-type grown mainly the Virginia-Carolina region at 10%. Climate of the Southeast is humid subtropical-like, with short/mild winters and long/hot summers allowing longer crop seasons but higher incidence of plant diseases.

Early leaf spot (ELS) caused by *Cercospora arachidicola* [Hori] and late leaf spot (LLS) caused by *Phaeoisariopsis personata* (also known as *Cercosporidium personatum* [Berk. & M.A. Curtis] Deighton) are serious fungal foliar diseases in the United States and around the world. Leaves with ELS symptoms generally exhibit brown lesions surrounded by a yellow ring on the upper side, while LLS symptoms show dark brown or black lesions on the underside of infected leaves [3]. Greater than 50% yield loss are observed for levels up to 95% defoliation, with ELS have been observed to be predominant in Virginia-Carolina region and LLS in the US Southeast region [4]. The Southeast weather exhibits high humidity, high temperature, and a long growing season (140–150 days) to maximize yield potential. To minimize general plant disease pressures, cultural practices such as crop rotation, weather prediction models coupled with fungicide applications, management of residue through tillage practices, and proper irrigation are deployed [5, 6]. Fungicide application to control leaf spot diseases is an effective method but can easily cost over $100 per acre and is not amenable to organic production. Therefore, the development of disease resistant peanut cultivars is a sustainable strategy for peanut production.

Identification of major genes for disease resistance has been very elusive based on strong environmental and genetic interactions and the involvement of multiple QTLs [7–9]. The nature of plant and fungal interactions makes visual selections highly variable based on years and locations. Several ELS resistant lines were produced from *A. cardenassii* (diploid) from initial resources [10, 11], including ICGV86699 and GPBD 4 from India [12] and GP-NC WS16 from North Carolina State U. [13] with some resistance to LLS. Han et al. [7] identified QTLs for ELS and LLS using single nucleotide polymorphism (SNP)-based linkage map, with analysis of interval sequences indicated a major QTL for LLS resistance. Chu et al. [8] identified 3 resistance QTLs on chromosome 3 and 1 on chromosome 5 evaluating recombinant inbred lines (RILs) from a population (Florida-07 × GP-NC WS16) segregating for resistance. Zhang et al. [9] identified 2 QTLs on chromosome B09 that were significantly associated with ELS and LLS resistance evaluating the US mini-core peanut collection. A high number of QTLs were identified, and the variability of field disease evaluations highlight the complexity of breeding for leaf spot resistance.

Innate immunity in plants is composed of a two-tier system for the defense against pathogens. At the site of infection, plants activate cell surface-localized membrane-associated pattern recognition receptors (PRRs) such as receptor like kinases (RLKs) or receptor like proteins (RLPs) that sense pathogen-associated molecular patterns (PAMPs) resulting in PAMP-triggered immunity (PTI) response [14]. The second line of defense involves the recognition pathogen avirulence (Avr) effectors by disease resistance (*R*) genes represented by (CNL) [coiled–coiled (CC), nucleotide-binding site (NBS), leucine rich repeat (LRR)] or (TNL) [Toll/interleukin-1 receptor (TIR) (NBS) (LRR)] that leads to pathogen specific effector-triggered immunity (ETI) [15]. *R*-genes have been identified and cloned in many plant species including *Arabidopsis thaliana*, *Glycine max*, *Medicago truncatula*, *Oryza sativa*, and *Triticum aestivum* via 5 classes of conserved motif, with the largest motif is nucleotide binding-leucine-rich repeats (*NBS-LRR*s) [15, 16].

A joint peanut breeding program between Auburn University, AL and the National Peanut Research Laboratory, Dawson, Ga was established to develop peanut varieties with desired agronomic traits for the peanut industry. One of a major goal is to develop disease resistant peanut varieties, currently focusing on leaf spot resistance, through plant selection and applications of genomics and molecular breeding strategies [9, 17]. A strategy is to develop several strategic crosses, followed by field performance studies, and evaluation of specific gene-expression to identify potential leaf spot resistance genes. Dang et al. [17] identified a set of 214 expressed *R*-genes in peanut leaves that were naturally challenged with leaf spot pathogens in peanuts, of which 76 were selected for gene-expression experiments based on visible PCR products on agarose gel electrophoresis and the successful testing of real time quantitative (q)PCR primers utilizing cDNAs from a susceptible and a tolerant peanut lines. The goal of this research is to identify *R*-gene expression that is correlated with leaf spot resistant peanut genotypes and to utilize gene-expression profiles and DNA polymorphisms for plant selection in future crosses.

## Materials and methods

### Genotype selection and disease rating

A total of 48 peanut genotypes, including 45 advanced breeding lines and 3 varieties (checks) with established responses to leaf spot diseases for comparison, were evaluated for leaf spot resistance. These peanut genotypes were mostly runner-type and have been derived from different crosses (Table 1). Peanuts were grown in the field (Dawson, GA) in the 2017 growing season using a randomize block design (RCBD) with 4 replications. Each row replications were 3 m in length with seeding rate at 20 seeds m^−1^ with 0.91 m between rows. Crop maintenance was according to best management practices with herbicide and insecticide, but no fungicide applications. Supplemental irrigation was provided as needed. Common disease symptoms for ELS were often dark brown spots surrounded by light yellow ring on the upper surface of the leaves, whereas symptoms for LLS are black spots on the underside of the leaves. Visual disease assessments for ELS and LLS were evaluated individually and defoliation percentages were assessed together using the Florida 1–10 scoring system at 130 days after planting (a week before harvest), where 1 = no disease and 10 = complete defoliation [18]. Leaves were randomly collected from nine different plants from each linear row replications. Three individual leaves were trimmed to proper weight between 0.2 and 0.3 g representing 1 technical replicate and 3 technical replicates were collected, placed into 2 mL homogenization tubes preloaded with 5 ceramic beads (28 mm), and placed into -80 °C freezer for later processing. A subset of peanut genotypes evaluated for leaf spot resistance was selected for real time quantitative (q)PCR analysis including 4 resistant lines, 3 varieties (checks), and 4 susceptible lines (Table 2).

**Table 1:**
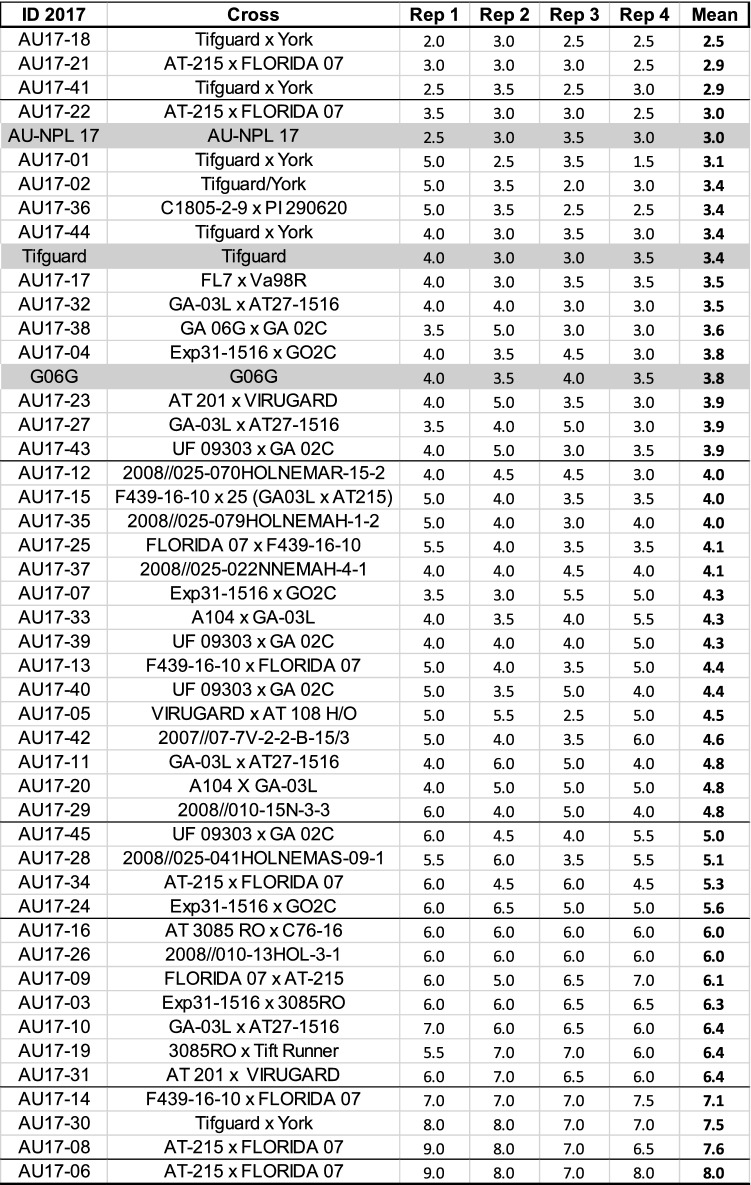
Peanut breeding lines, pedigrees, and leaf spot ratings

**Table 2:** Peanut genotype selection for real time qPCR applications

ID 2017	Cross	Rep-1	Rep-2	Rep-3	Rep-4	LS mean
AU17-18	Tifguard/York	2	3	2.5	2.5	2.5
AU17-21	FLORIDA 07 X AT-215	3	3	3	2.5	2.9
AU17-41	Tifguard/York	2.5	3.5	2.5	3	2.9
AU17-22	FLORIDA 07 X AT-215	3.5	3	3	2.5	3
AU-NPL 17	AU-NPL 17	2.5	3	3.5	3	3
Tifguard	Tifguard	4	3.5	4	4	3.4
G06G	G06G	4	4	3	3	3.8
AU17-14	F439-16–10 X 25 (GA03L x AT215)	7	7	7	7.5	7.1
AU17-30	Tifguard/York	8	8	7	7	7.5
AU17-08	FLORIDA 07 X AT-215	9	8	7	6.5	7.6
AU17-06	FLORIDA 07 X AT-215	9	8	7	8	8

### RNA extraction

Total RNAs were extracted from fresh-frozen leaves utilizing Trizol Reagent and PureLink RNA mini kit (Invitrogen). Trizol LS was freshly diluted 1:4 with DEPC water (HB) and cooled on ice before use. An aliquot of 500 µL of HB was added to the frozen tissue on ice and immediately homogenized utilizing a Bead Ruptor Elite (Omni International, Atlanta, GA) at a setting (S = 6.00, T = 0:45, C = 02, D = 0:10), and homogenized samples were immediately placed on ice for 5 min. Another aliquot of 500 µL HB was added, shaken rigorously for 5 min., then placed on ice for 2 min. Then 200 µL chloroform was added, mixed well by physical inversion for 5 min, and placed on ice for 5 min. Samples were centrifuged (4 °C) 5 min at max speed (14,000–21,000 g). Purified aqueous solution (~ 700 µL top layer) was transferred to a new 2 mL tube and 1 volume (~ 700 µL) 70% room temperature ethanol was added and gently mixed. Supernatant was transferred to PureLink RNA purification column and processed according to manufacturer’s instruction. RNAs were quantified by nanodrop 2000 spectrophotometer (Thermo Fisher Sci., Waltham, MA) and quality was visualized by agarose gel electrophoresis.

### cDNA synthesis, standard and qPCR

Two micrograms of DNased treated total RNAs were utilized as template and cDNA synthesis was as described by Dang et al. [19]. Primers were designed utilizing Clone Manager (Sci-Ed Software, Denver, CO) for standard polymerase chain reaction (PCR) product analysis (Online Resource 1) and qPCR (Online Resource 2). Primers for qPCR were evaluated for functionality and details are described (Online Resource 2). Melt curve analysis showed single peaks for all primers evaluated indicating single PCR products. cDNAs were diluted 1:10 with diethyl pyrocarbonate (DEPC) treated water and utilized as starting PCR template. For standard PCR reactions, each sample mix consisted of 4 µL of diluted cDNAs, 1 µL (0.5 mM) of each specific forward and reverse primers, 10 µL GoTaq Green Master mix (Promega), and sterile water to a total of 20 µL volume. PCR cycling conditions were as follow: 1 cycle (2 min at 94 °C) to denature cDNAs, then 40 cycles (20 s at 94 °C, 20 s at 55 °C and 50 s at 72 °C), and 1 cycle (10 min at 72 °C) to complete PCR product synthesis. qPCR analysis was performed on a QuantStudio7 Flex real-time PCR system (Thermo Fisher Sci.) utilizing Relative Quantitation (RQ) as described by manufacturer. Three technical replicates were performed for each peanut genotype and *R*-gene combinations. A 20 µL total reaction mix consisted of 4 µL of diluted cDNAs, 1 µL (0.5 mM) of each forward and reverse specific primers, 10 µL PowerUp SYBR green master mix (Thermo Fisher Sci.), and 2 µL of sterile water. Cycling conditions consisted of 1 cycle each (2 min at 50 °C and 10 min at 95 °C), followed by 40 cycles (15 s at 95 °C and 1 min at 58 °C), and a dissociation curve analysis cycle (15 s at 95 °C, 20 s at 58 °C and 15 s at 95 °C). The threshold cycle (Ct) was generated using QuantStudio Real-Time PCR software (Thermo Fisher Sci.) and relative quantification (RQ) values were calculated based on 2^−∆∆Ct^ described by Livak and Schmittgen [20]. All samples were first normalized to *Actin* (EZ723877) as an internal control then transformed data were normalized with a susceptible line (AU17-14) 2^−∆∆Ct^ values and compared with the other 10 peanut genotypes to determine relative fold changes in gene-expression and graphed from the highest RQ level to the lowest for visual comparisons.

### PCR product generation, cloning, and sequencing

Standard PCR products were generated using cDNAs from a leaf spot susceptible (AU17-08) and a resistant line (AU17-41) and resolved by gel-electrophoresis (1% TAE). Visible bands were cut out from gels and purified using QIAquick Gel Extraction Kit (Qiagen, Valencia, CA) and concentration was determined using Nanodrop 2000 spectrophotometer (Thermo Fisher Sci.). Approximately 70 ng of purified-PCR products were sequenced utilizing dideoxy DNA sequencing method (Eurofins MWG Operon, Louisville, KY) with the specific forward or reverse primers. Purified PCR products were cloned using StrataClone PCR Cloning Kit according to manufacturer’s instruction (Stratagene, San Diego, CA). Plasmids were processed using QIAprep Spin Miniprep kit (Qiagen) and ~ 300 ng of purified plasmids were sequenced with T3 or T7 promoter sequencing primers (Eurofins MWG Operon, Louisville, KY, USA). Amplicon sizes for PCR fragments or cloned products were verified by sequencing (Table 3).

**Table 3:** Differential expressed *R*-genes, relative RQ fold differences comparing susceptible and resistant lines, chromosomal locations, putative gene functions, and SNPs within PCR product sizes

Target	Fold	Chrom#	Gene function identification	SNPs	Size
RGA020	− 3.27	5	Serine/threonine-protein kinase PBL19	0	886 bp
RGA023	− 1.254	4	Serine/threonine-protein kinase PBS1	0	1236 bp
RGA035	− 1.47	3	Serine/threonine-protein kinase	7	895 bp
RGA054	− 1.48	1A, 10B	LRR receptor-like serine/threonine-protein kinase FEI 1	10	1503 bp
RGA055	− 1.90	5	Serine/threonine-protein kinase CST	0	1031 bp
RGA060	− 2.76	3	TMV resistance protein N	0	526 bp
RGA068	− 1.23	4	TMV resistance protein N-like	15	573 bp
RGA078	− 1.39	1	Mitogen-activated protein kinase kinase 2	6	920 bp
RGA099	− 1.30	5A, 4B	Serine/threonine-protein kinase PBL2	0	1013 bp
RGA107	− 1.55	8	Phytosulfokine receptor 2	13	1263 bp
RGA113a	− 1.60	2	Serine/threonine-protein kinase PBL7	0	865 bp
RGA124	− 2.84	8	DNA damage-repair/toleration protein DRT100	4	889 bp
RGA147b	− 1.02	1	Serine/threonine-protein kinase STY13	7	1013 bp
RGA153b	− 1.96	10	Receptor-like serine/threonine-protein kinase SD1-8	3	596 bp
RGA166	− 1.29	10	Receptor-like serine/threonine-protein kinase RPK2	13	1553 bp
RGA172	− 1.57	10	Serine/threonine-protein kinase HT1	0	1043 bp
RGA179	− 2.32	3A, 8B	Serine/threonine-protein kinase CTR1	2	1570 bp
RGA199	− 1.63	1	Serine/threonine-protein kinase PIX7	12	1104 bp
RGA207	− 1.01	6	Receptor-like serine/threonine-protein kinase RPK2	0	1366 bp
RGA226	− 2.47	3	L-type lectin-domain containing receptor kinase IX.1-like	4	727 bp
RGA237	− 1.66	2	Serine/threonine-protein kinase CTR1-like	2	1911 bp
RGA238	− 2.54	6	Serine/threonine-protein kinase PBL19	0	1044 bp
RGA246	− 0.95	3A, 8B	Leucine-rich repeat receptor-like protein kinase At2g33170	8	1935 bp
RGA249a	− 2.32	3	Serine/threonine-protein kinase PBL19	6	1117 bp
RGA253	− 2.42	8	Disease resistance-like protein DSC1	0	460 bp
RGA255	− 1.51	10	Receptor-like protein kinase HAIKU2	22	1390 bp
RGA265	− 1.31	4B	TMV resistance protein N	5	730 bp
RGA286	− 1.11	5	Serine/threonine-protein kinase PIX7	5	1228 bp
RGA304	− 3.69	5	Receptor-like serine/threonine-protein kinase BAM1	1	1516 bp
RGA314	− 0.95	8A, 7B	Receptor-like protein kinase HSL1	5	1503 bp
RGA318	− 1.25	4	TMV resistance protein N	1	731 bp
RGA336	− 1.71	4	Mitogen-activated protein kinase homolog MMK2	1	941 bp
RGA360	− 3.88	1	Receptor-like protein kinase At5g18500	0	1304 bp
RGA365	− 2.23	1	U-box domain-containing protein 34	0	1470 bp
RGA366	− 2.50	8A, 10B	Receptor-like serine/threonine-protein kinase At1g74360	12	1523 bp
RGA369	− 3.72	8A, 7B	Receptor-like serine/threonine-protein kinase At5g57670	8	963 bp

### SNP determination, mapping to subgenomes, and gene function identity

Sequencing analysis was performed using Sequencher (Gene Codes, Ann Arbor, MI) and visual observation of sequencing peaks within each sequence verified the locations of single nucleotide polymorphisms (SNPs). Mapping to subgenomes was performed using Blastn (NCBI) to *Arachis duranensis*, *A. ipaensis* diploid genotypes, and *A. hypogaea* tetraploid genomes. Putative functions were determined by motif searches using Blastx (NCBI) and HMMER (pfam.xfam.org).

## Results

### Disease ratings and classifications

**Fig. 1:**
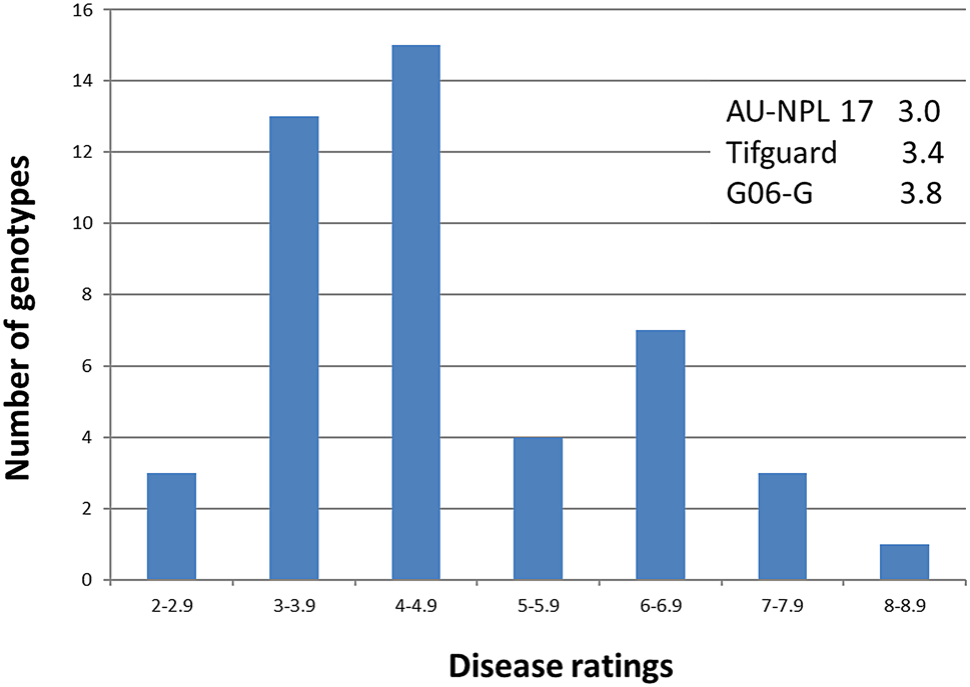
Distribution of leaf spot disease ratings among the 45 peanut breeding lines. Varieties (checks) disease ratings were included for comparison

Leaf spot visual ratings for the 48 peanut genotypes were based on scale of 1 to 10 with 1 having no visible symptoms and 10 having complete defoliation. The 45 peanut breeding lines (Table 1) with different pedigrees were separated into seven groups (Fig. 1) from the lowest to the highest disease severity: (1) 2.0–2.9 (3), (2) 3.0–3.9 (12), (3) 4.0–4.9 (15), (4) 5.0–5.9 (4), (5) 6.0–6.9 (7), (6) 7.0–7.9 (3), (7) 8.0–8.9 (1), and the 3 variety checks belonging to group 2 with disease ratings between 3.0 to 3.9. A subset of peanut lines was selected for qPCR analysis. Four breeding lines with low disease ratings between 2 and 3 (AU-18, AU-21, AU-41, AU-22), 3 peanut varieties (AU-NPL 17, Tifguard, G06G), and 4 lines with high disease ratings between 7 and 8 (AU-14, AU-30, AU-08, AU-06) were selected for *R*-gene expression studies (Table 2). AU-21 and AU-22 (low disease) contrast with AU-06 and AU-08 (high disease) that share the same pedigree AT-215 × Florida 07. Similarly, AU-18 and AU-41 (low disease) contrast with AU-30 (high disease) with the same pedigree of Tifguard x York.

### *R*-gene expression

**Fig. 2:**
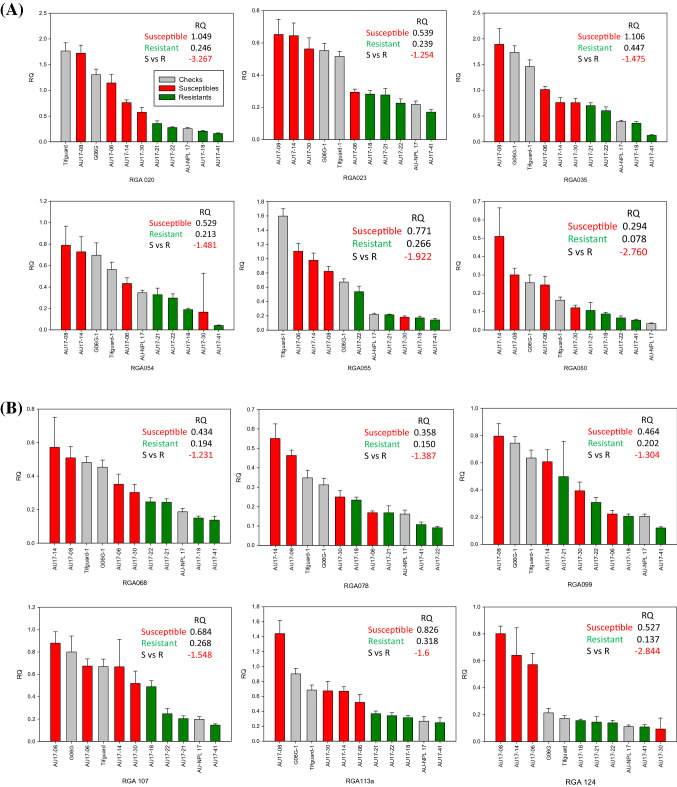

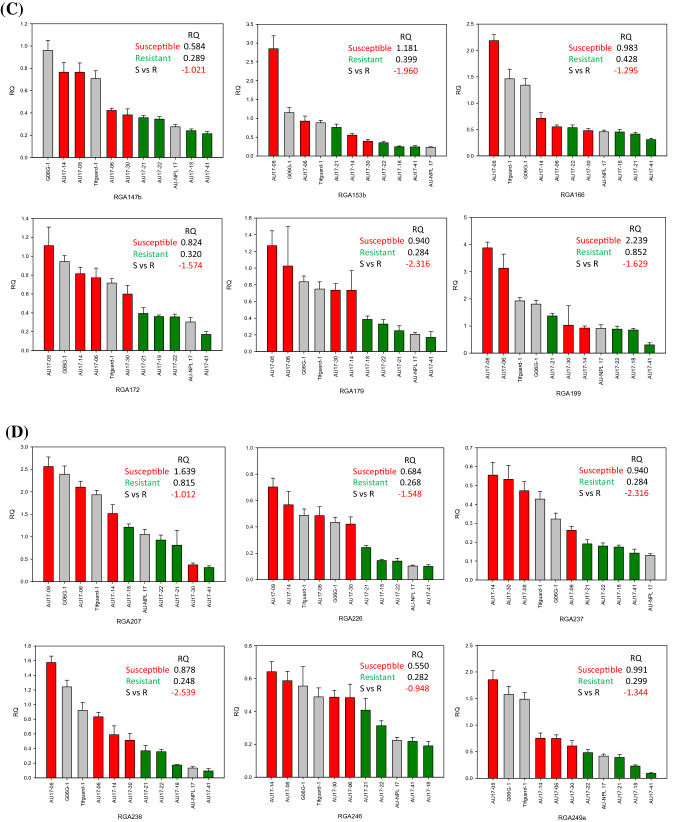

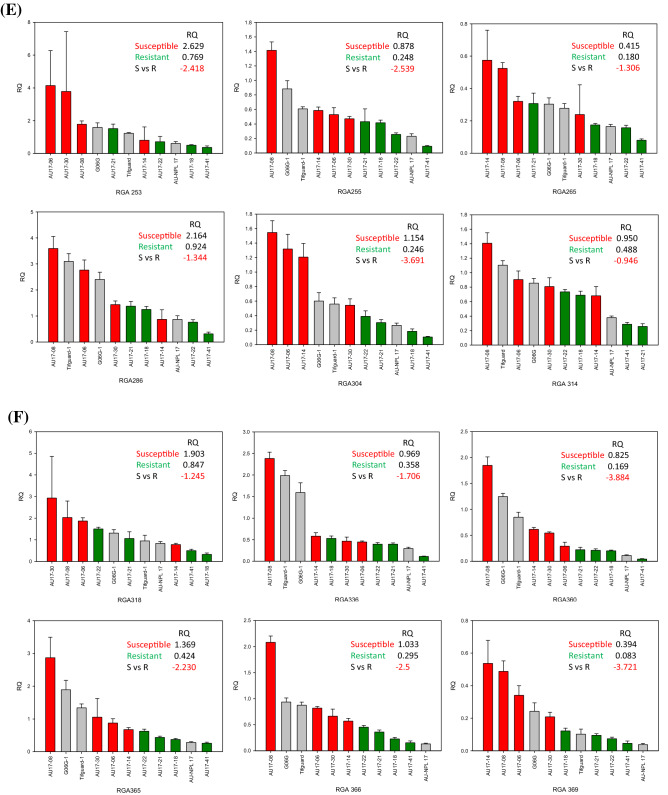
**a**-**f** Relative Quantification (RQ) of gene-expression levels comparing susceptible to resistant lines graphed from left to right. Checks were included for comparison. Relative fold differences (negative correlation) were the average of resistant line RQs divided by susceptible line RQs. Panels A through F showed 36 differentially regulated *R*-genes in sequential order separating susceptible lines (left) from resistant lines (right)

A total of 76 *R*-genes were evaluated in the selected peanut genotypes from the set of 89 *R*-genes that were evaluated by Dang et al. [17]. Relative quantitative (RQ) gene-expression levels were compared between susceptible lines to resistant lines. Varieties (checks) were also included for reference. The levels of gene-expression for the selected genotypes were graphed to observe correlations with leaf spot resistance. A set of 36 *R*-genes were differentially regulated separating susceptible lines (left side) away from resistant lines (right side) (Fig. 2). RQs from 4 susceptible lines were averaged and divided by the average of RQs from 4 resistant lines to generate fold differences, showing that all 36 *R*-genes were negatively correlated comparing susceptible to resistant lines ranging from − 3.88 to − 0.95. This was further divided into 3 groups: 1) all four susceptible lines clustered from the 4 resistant lines which included RGAs 020, 023, 035, 060, 068, 107, 113a, 147b, 172, 179, 226, 237, 238, 246, 249a, 255, 304, 360, 365, 366, and 369; 2) one susceptible line clustered with 4 resistant lines which included RGAs 054, 055, 078, 124, 166, 207, 253, 265, 286, 314, and 318; 3) 2 susceptible lines clustered with 4 resistant lines which included RGAs 099, 153b, 199, and 336. Out of the 3 variety checks, Tifguard and G06G consistently clustered with susceptible lines and AU-NPL 17 clustered with resistant lines. From most of the graphs, AU17-08 was observed to be on the extreme left of the chart indicating the most susceptible and AU-41 on the extreme right as the most resistant.

### Chromosome locations and *R*-gene putative functions

*R*-genes were mapped to the reference peanut genomes of diploid peanut progenitors *Arachis duranensis* and *A. ipaensis* genotypes, representing the A and B subgenomes of peanut [26] and cultivated tetraploid peanut (*A. hypogaea*) [16]. The 36 *R*-genes were mapped to most of the peanut diploids and tetraploid subgenomes, except for chromosome 09 (Table 3). RGAs 078,147b, 199, 360, and 365 were mapped to both chromosomes A01 and B01, with RGA 054 on A01 and B10; RGAs 113a and 237 were on A02 and B02; RGAs 035, 060, 226, and 249a were on A03 and B03, with RGAs 179 and 246 on A03 and B08; RGAs 023, 068, 318, and 336 were on A04 and B04, with RGA 265 on only B04 and RGA 099 on both B04 and A05; RGAs 020, 055, 286, and 304 were on A05 and B05; RGAs 207 and 238 were on A06 and B06; RGAs 314 and 369 were on both B07 and A08; RGAs 107, 124, and 253 were on A08 and B08, with RGA 366 on both A08 and B10; no RGAs were on A09 or B09; RGAs 153b, 166, 172, 255 were on A10 and B10. Blastx (NCBI) and HMMER (Pfam) database searches revealed potential function of the candidate *R*-genes (Table 3). Of the 36 *R*-genes evaluated, 29 were classified as RLKs and 3 RLPs which are part of PTI response through pattern-recognition of the pathogen by the host. Four RGAs (060, 068, 265, and 318) are TNLs that have homology to TMV resistance protein N originally studied in tobacco that showed resistance to tobacco mosaic virus. A ranged of observed SNPs (0 to 22) by the PCR product sequencing size indicate multi-allelic possibilities.

## Discussions

ELS and LLS are major diseases of peanuts and the range of severity is strongly influenced by G x E associated with many QTLs [21–23]. Only a few peanut genotypes have been identified as medium tolerant to leaf spot diseases and specific crosses were generated to develop more tolerant lines [8]. Marker types, such as simple sequence repeats (SSRs) and Insertions/Deletions (Indels), were applied to select leaf spot resistant lines [24, 25], but validation has been difficult due to variations in leaf spot disease phenotyping (strong environmental effect, different levels of pathogen pressure, state of plant health). For example, LLS have been observed more frequently in the Southeast US and ELS are more prominent in the Carolina regions [4].

Disease resistance (*R*) genes play a major role in response to pathogen infection in plants. Candidate *R*-genes have been identified on the diploid progenitors of the cultivated peanut, 345 in the *Arachis duranensis* and 397 in the *A. ipaensis* genotypes, representing the A and B subgenomes of peanut [26]. Recently, 713 candidate *R*-genes were identified in the cultivated peanut (*A. hypogaea*) tetraploid genome [16]. Because of the high potential that *R*-genes may be involved in ELS and LLS resistance in peanuts, several research groups attempted to correlate the identification of leaf spot resistant QTLs with candidate *R*-genes within a proximal genomic location. Zhou et al. [21] evaluated a recombinant inbred line (RIL) population derived from Zhonghua x ICGV 86,699 in 3 different environments in multiple years. Significant G x E interaction was observed and multiple QTLs for LLS resistance were identified. A major QTL *qLLSB6-7* was located proximal to six NBS-LRR encoding genes covering 3.9 Mb and another QTL *qLLSB1* was identified on chromosome B01 containing five NBS-LRR encoding genes that covered an 8.9 Mb segment. Han et al. [7] identified QTLs for ELS and LLS using SNP-based linkage map, with analysis of interval sequences indicated a major QTL for LLS resistance was flanked by two NBS-LRR resistance genes on chromosome B05 and two homologs of TMV resistance protein N for ELS was revealed on chromosome A03. Shirasawa et al. [27] identified a major QTL for LLS resistance on chromosome A02 evaluating RILs from a TAG24 x GPBD 4 cross. *R*-gene candidates were identified within the proximal genomic region, including 2 LRR and NB-ARC domain proteins, a TIR-NBS-LRR domain resistant protein, and an MLO-like protein. Chu et al. [8] revealed 3 resistant QTLs on chromosomes A05, B05, and B03 for LLS and 3 on chromosomes A03 and B03 for ELS, evaluating RILs from a Florida-07 x (GP-NC WS16) cross. Candidate *R*-genes with NBS-LRR motifs and threonine-protein phosphatases were within proximal genomics location to QTL *qLLS.A03* locus covering a range of 95 to 132 Mb segment. Zhang et al. [9] identified 2 QTLs on chromosome B09 that are significantly associated with ELS and LLS resistance evaluating the US mini-core peanut collection. Candidate *R*-genes include TIR-NBS-LRR class, LRR family proteins, and putative disease resistance RPP13-like proteins within proximal distance form QTLs. QTLs identified in these studies reportedly do not overlap since different RIL populations were evaluated and in different growing environments.

At the site of fungal infection on the cell surface, pathogen-associated molecular patterns (PAMPs) come into contact with immune receptors of the plant usually associated with leucine-rich repeat receptor on the extracellular side anchored in cell membrane by transmembrane domain and either a kinase (RLKs) or protein (RLPs) on the intracellular side. Out of the 36 *R*-genes identified to be differentially regulated in resistant lines, 29 are RLKs and 3 are RLPs. All 36 *R*-genes are expressed at significantly lower levels than susceptible lines. Recognition of PAMP of pathogens by RLKs and RLPs leads to activation of downstream signaling including calcium influx, a rapid burst of reactive oxygen species (ROS), activation of mitogen-activated protein kinase (MAPK) cascades, regulation of calcium-dependent protein kinases (CPKs), transcriptional activation, and phytohormone regulation [28]. Receptor-like cytoplasmic kinases (RLCKs) VII are part of all of plant receptor-like kinases that do not contain extracellular and transmembrane domains, and RGA020 (PBL19), RGA023 (PBS1), RGA099 (PBL2), RGA113a (PBL7), RGA238 (PBL19), RGA 249a (PBL19) have homology to this group. In *Arabidopsis* there are 46 members, and some have been identified to be important as positive regulators in PTI signaling responses in pathogen attack, with some members having the same or similar function to complement each other [29]. A unique member, *PBL13*, acts as a negative regulator in pattern-induced reactive oxygen species (ROS) to ensure the proper activation and signaling to pathogen invasion [30]. The recognition of microbial or fungal pathogens followed by a hypersensitive response is a mechanism that stops the spread of pathogen. RGA226 codes for a L-type lectin-domain containing receptor kinase IX which was shown to be involved in *Phytophora* and induction of programmed cell death in *Arabidopsis* [31]. Hormone regulation and modulation of cell structure is part of the immune response. RGA054 has homology to LRR receptor-like serine/threonine protein kinase FEI that has shown to be important in regulating cell wall function through 1-aminocyclopropane-1-carboxylic acid (ACC) synthase-mediated signal [32]. Also, RGA107 codes for a phytosulfokine receptor that disruption or overexpression of this gene affects cellular growth and longevity [33]. Repair mechanism after cell damage may be important. RGA124 matched to a DNA-damage-repair/toleration protein which plays an important role in the repair and tolerance of UV-B induced DNA damage [34]. Four *R*-genes (RGA 060, 068, 265, 318) were homologous to TMV resistant protein N which has shown to confer resistance against tobacco mosaic virus (TMV) in tobacco and mutations to any of the conserved motifs TIR-NBS-LRR will reduce either sensitivity of pathogen recognition, induction of hypersensitive response, or movement of TMV throughout the plant [35]. RGA068 was highly polymorphic with SNP ratio (15 in 573 bp), and PCR cloning and sequencing result showed 6 different protein product variants when aligned at 98% identity. A high number of sequence variations indicating many alleles for TMV N-like proteins in peanut may recognize common Avr proteins in different pathogens [36].

At the molecular level, leaf spot resistance is associated with differential gene-expression involving many biological pathways comparing mutant and wild-type peanut genotypes in response to LLS [37]. Furthermore, DNA methylation and gene-expression analysis revealed the epigenetics regulation for leaf spot resistance in peanut [38]. *R*-gene expression has been positively correlated with disease resistance under high pathogen load but can have a negative effect of growth and development presumedly because of metabolic costs [39]. *R*-gene expression variations were observed to emulate the patterns of environmental conditions, such as humidity and temperature, that is conducive to the disease [40]. In terms of *R*-gene evolution in peanuts, Song et al. [16] observed that a majority (727 out of 756) *R*-gene candidates in the tetraploid genome (cv Tifrunner) are recently produced resulting from gene duplication events after tetraploidation. RLKs and RLPs, representatives of all *R*-genes, are of PAMP pathway which allows broad-spectrum PTI to various pathogens. The presence of multiple QTLs and high G x E components make breeding for leaf spot resistance very challenging. The identification and association of *R*-gene candidates to leaf spot resistance will facilitate the development of molecular markers that can be applied to future crosses.

## Supplementary Information

Below is the link to the electronic supplementary material.

Supplementary file1 Standard PCR primers and their respected PCR product size (XLSX 15 KB)

Supplementary file2 Quantitative (q)PCR primers and their respected PCR reaction efficiencies (XLSX 12 KB)
